# Precision scalpels for the epigenome: next-gen editing tools in targeted therapies

**DOI:** 10.3389/fmed.2025.1613722

**Published:** 2025-06-17

**Authors:** Ruiming Zhang, Tianqi Yao, Meiyin Fan, Xiaoying Jiang, Keshan Wang, Min Cui, Kaijian Bing, Xiaotian Xia

**Affiliations:** ^1^Department of Nuclear Medicine, Union Hospital, Tongji Medical College, Huazhong University of Science and Technology, Wuhan, China; ^2^Tongji Medical College, Huazhong University of Science and Technology, Wuhan, China; ^3^Health Management Center, Union Hospital, Tongji Medical College, Huazhong University of Science and Technology, Wuhan, China; ^4^Department of Urology, Union Hospital, Tongji Medical College, Huazhong University of Science and Technology, Wuhan, China; ^5^Department of Orthopaedics, Union Hospital, Tongji Medical College, Huazhong University of Science and Technology, Wuhan, China; ^6^Department of Surgical Education Research, Union Hospital, Tongji Medical College, Huazhong University of Science and Technology, Wuhan, China; ^7^Hubei Province Key Laboratory of Molecular Imaging, Wuhan, China

**Keywords:** gene editing, epigenetic editors, cancer target therapy, metabolic disease, neurological diseases

## Abstract

This article reviews the fundamental concepts of epigenetics and its related mechanisms, and discusses recent advances in epigenetic gene editors and their applications in disease treatment. First, the article introduces the concept of epigenetic inheritance and the four main epigenetic mechanisms. Then, after briefly outlining traditional gene editing, it presents epigenetic gene editors, their associated tools, and the historical context of their development. Subsequently, the article describes the working principles and advantages of epigenetic editing tools such as KRAB and DNMT. Addressing the current bottlenecks in the field, the article provides an in-depth analysis of editing efficiency and specificity, long-term safety, and the complexity of clinical applications. In addition, it discusses optimization strategies for delivery systems, minimization of off-target effects, and therapeutic approaches for multigene disorders. Finally, the article outlines the progress of epigenetic editors in both neoplastic and non-neoplastic disease research. In summary, this article offers a comprehensive review of the theoretical foundations of epigenetics, the evolution of gene editing tools, and the latest advances in epigenetic editors for disease treatment, providing a valuable reference for future research and clinical application.

## 1 A brief description of epigenetics

Epigenetics is the study of heritable changes in gene function that occur without alterations to the DNA sequence. Such changes are caused by a combination of environmental factors and genetic material, and can be transmitted to offspring through both mitosis and meiosis. In contrast to classical genetics-which focuses on changes in gene expression resulting from sequence alterations such as mutations, loss of heterozygosity, and microsatellite instability-the mechanisms of epigenetics primarily involve covalent modifications of DNA, covalent modifications of proteins, chromatin remodeling, and regulation by non-coding RNAs. These mechanisms regulate gene expression and influence biological phenotypes (1).

## 2 Mechanisms of epigenetic inheritance

There are varieties of forms and mechanisms in epigenetic inheritance and its regulation. Here we choose some most common ones to introduce.

### 2.1 Covalent modification of DNA

The best known and most important form of DNA modification is DNA methylation, which is widespread in plant and animal cells and bacteria. DNA methylation refers to the process of covalent attachment of methyl groups to specific bases in the DNA molecule under the action of DNA methylation transferase enzymes (DNMTs). DNA methylation is a form of chemical modification of DNA, which is a highly conserved epigenetic modification that alters the activity of DNA fragments, and thus the genetic expression of the DNA fragments, without altering the sequence of the DNA. DNA methylation can alter the activity of DNA fragments without altering the DNA sequence, changing genetic expression, and is a very conservative epigenetic modification. DNA methylation can occur at the C-5 position of cytosine, the N-6 position of adenine, the N-7 position of guanine, etc., which are catalyzed by various DNA methylation enzymes to produce 5-methylcytosine (5-mC), N6-methyladenine (N6-mA), and 7-methylguanine (7-mG), of which cytosine methylation has been the most extensively studied (2). When DNA methylation occurs, cytosine protrudes from the DNA double helix and enters the cleft where it can bind to the enzyme and, catalyzed by cytosine DNMTs, the active methyl group is transferred from the S-adenosylmethionine to the cytosine 5 position, resulting in the formation of 5-methylcytosine (5-mC). DNA methylation induces changes in chromatin structure, DNA conformation, DNA stability and the way DNA interact with proteins, thereby controlling gene expression, and plays a crucial role in maintaining normal cell function, X chromosome inactivation in females, genome structural stability, genetic imprinting, embryonic development, and the onset and development of tumors and disease. In addition to DNA methylation, covalent modifications of DNA include DNA glycosylation, DNA oxidation, DNA adduct formation and other modalities (3, 4).

### 2.2 Covalent modification of protein

Protein covalent modification is the process by which proteins are covalently attached to other molecules through chemical reactions, resulting in changes in their physicochemical properties and functions. Such modifications extend the chemical composition and information of the 20 natural amino acids and have a significant impact on proteins, including activity, stability, function, structure, localization, trafficking, signaling and their mode of interaction with partner biomolecules. Covalent modifications of proteins can be broadly divided into covalent modifications of histones and covalent modifications of non-histone proteins. Of these, covalent modification of histones is the most dominant. Histones are the basic structural proteins of eukaryotic chromosomes and play a crucial role in the process of DNA folding to form chromatin. In addition to the induction of transcription factors and hypomethylation of promoter regions, the activation of histone modification sites is required for normal gene expression (5). Covalent modifications of histones are diverse and include the presence of phosphorylation and dephosphorylation, methylation and demethylation, ubiquitination, and deubiquitination. Among these, phosphorylation and dephosphorylation of proteins are the most common and important post-translational modifications. This reversible mechanism is mediated by protein kinases that convert proteins from hydrophobic non-polar to hydrophilic polar, allowing proteins to change conformation when interacting with other molecules. Phosphorylated amino acids can bind molecules capable of interacting with other proteins, thereby assembling and disassembling protein complexes (6). In addition, ubiquitination and deubiquitination are protein modification methods that have been studied extensively in recent years. Ubiquitin is a highly conserved small protein of 76 amino acid residues (molecular weight approximately 8.5 kDa) that is present in almost all eukaryotic tissues. Ubiquitination is a very important post-translational protein modification process in eukaryotic cells, and its most important function is to target substrate proteins for degradation by the 26S proteasome, in addition to being involved in a variety of functions such as cellular signaling, cell cycle regulation, DNA repair and other functions by altering the structure, function, localization and assembly of proteins (7).

### 2.3 Chromatin remodeling

Chromatin remodeling refers to the regulation that affects gene expression by altering the structure and composition of chromatin, which plays a critical role in the regulation of epigenetic inheritance (8). Chromatin remodeling is mainly mediated by ATP-dependent protein complexes or covalent modification of histone tails by histone modifying proteins (e.g., Polycomb histones, which is also known as PcG proteins, including Polycomb Repressor Protein complexes, PRCs) to silence or activate gene expression (9).

#### 2.3.1 ATP-dependent chromatin remodeling

Common ATP-dependent complexes in eukaryotes include SWItch/Sucrose Non-Fermentable (SWI/SNF), Imitation SWItch (ISWI), Chromodomain helicase DNA-binding proteins (CHD), and INO80 complex (chromatin remodeling ATPase). SWI/SNF affects transcription levels by binding to and dissociating nucleosomes from DNA, generating transient DNA loops that move around the nucleosome, allowing nucleosome repositioning and facilitating transcriptional activation or repression, depending on whether the target gene is located in an open chromatin or compact chromatin region. The ISWI complex affects the level of transcription by altering the distance and stability between nucleosomes; the CHD complex affects the active state of genes by recognizing specific chromatin markers (e.g., methylated H3K4 or acetylated H3K9, etc.) and by altering the localization of nucleosomes on DNA through energy-driven changes generated by ATP hydrolysis.

#### 2.3.2 PcG proteins and chromatin remodeling

PcG proteins are widely involved in gene silencing during cellular differentiation and play important roles in chromatin remodeling and epigenetic regulation through the formation of multi-protein complexes (i.e., PRCs), PRCs can catalyze specific histone modifications to maintain the silenced state of genes, for example, they catalyze the trimethylation of histone H3K27, H3K37me3 is a strong transcriptional repressor mark; in addition, PRCs can catalyze the monoubiquitination of H2AK119, further stabilizing the compact state of chromatin. Thus, there are complex interactions and co-ordination mechanisms between chromatin remodeling and epigenetic modifications such as DNA methylation and histone modifications, which are involved in physiopathological processes such as embryonic development, cancer, cardiovascular diseases and neurodegenerative diseases (10, 11).

### 2.4 Regulation of non-coding RNAs

Non-coding RNA is a class of functional RNA molecules that are not translated into proteins and is divided into structural non-coding RNAs and functional non-coding RNAs, the former including the well-known rRNAs (ribosomal RNAs) and tRNAs (transfer RNAs), and the latter is divided into long non-coding RNAs and short non-coding RNAs according to the length of the nucleotides, of which the most hotly debated and intensively researched are long non-coding RNAs (lncRNAs), circular RNAs (circRNAs) and microRNA (miRNAs) (12–14). MiRNA is a class of short endogenous non-coding RNAs that can regulate the effect of gene expression at the post-transcriptional level by inhibiting translation or degrading mRNA. An increasing number of studies have shown that miRNAs play an important role as regulatory elements in the regulatory mechanisms of various organisms (15, 16). CircRNAs are single-stranded, covalently closed RNA molecules whose mechanisms of action can be broadly categorized into the following three types: (1) Regulation of gene transcription: Regulation of gene expression at the transcriptional level by complementary base pairing. (2) MicroRNA sponges: MicroRNA sponges are artificial RNA structures designed to regulate miRNA levels, with multiple miRNA binding sites capable of adsorbing and reducing the effective concentration of specific miRNAs, thereby interfering with the regulatory effects of miRNAs on their target genes. circRNAs act as miRNA sponges: As endogenous binding RNAs, circRNAs act as miRNA sponges that indirectly regulate the expression of target genes downstream of miRNAs. (3) Protein scaffolds: circRNAs can bind to proteins with different functions and play the functions of inhibiting protein activity, recruiting components of protein complexes, or regulating protein activity, etc. (17). LncRNAs are a class of long non-coding RNAs transcribed by RNA polymerase, and studies have shown that they are involved in a variety of biological processes, including reprogramming of pluripotent stem cells, oncogenic progression, and cell cycle regulation. The mechanism of lncRNAs can be simply summarized as the following four: (1) Signaling: Numerous studies have shown that under different stimulus conditions and signaling pathways, lncRNAs are specifically transcribed and participate in specific signaling pathways as signal transducing molecules. (2) Decoy: Once transcribed, this type of lncRNA can bind to DNA-binding proteins (e.g., transcription factors), blocking the action of protein molecules and regulating the expression of downstream genes. (3) Guide: This type of lncRNA binds to proteins (usually transcription factors) and then localizes the protein complex to specific DNA sequences to regulate gene expression. (4) Scaffold: lncRNAs act as a “central platform” that allows two or more proteins to bind to the lncRNA molecule to form a complex. In the cell, when multiple signaling pathways are activated simultaneously, these downstream effector molecules can bind to the same lncRNA molecule, enabling convergence and integration of information between different signaling pathways (18, 19).

**FIGURE 1:**
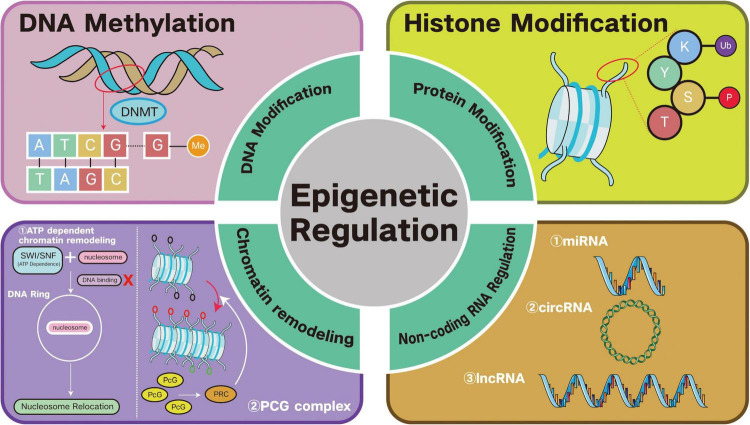
Four mechanisms of epigenetic inheritance: (1) covalent DNA modifications, such as DNA methylation mediated by DNA methyltransferase (DNMT); (2) covalent protein modifications, such as various types of histone modifications; (3) chromatin remodeling, mainly mediated by ATP-dependent protein complexes or covalent modifications of histone tails by histone-modifying proteins (e.g., Polycomb histones, which is also known as PcG proteins, including Polycomb Repressor Protein complexes, PRCs); (4) non-RNA regulation, mainly involving microRNA (miRNA), circular RNA (circRNA), and long non-coding RNA (lncRNA). These four mechanisms are widespread in organisms and work together to regulate gene expression.

The common mechanisms of epigenetic inheritance is shown and summarized in Figure 1, and Table 1 shows the different effects on genes of various mechanisms of epigenetic inheritance.

**TABLE 1:** Summary of gene regulatory effects associated with various mechanisms of epigenetic inheritance.

Type	Modification (examples)	Effect	Mechanism	Related diseases
DNA modifications	High methylation of CpG islands in the promoter region	Inhibition	DNMT catalyzes methylation, block transcription factor binding, and recruit methyl-binding proteins (e.g., MBD).	Breast cancer, colorectal cancer
	Hypomethylation of CpG islands in promoter region	Activation	TET catalyzes the conversion of 5 mC–5 hmC, relieving transcriptional repression and enhancing chromatin accessibility.	Dysplasia
Protein modifications	Ubiquitination (H2AK119ub1)	Inhibition	PRC1 complex catalyzes and recruits PRC2 to mediate the deposition of H3K27me3, maintaining heterochromatin silencing.	Leukemia, solid tumor
	Phosphorylation (H3S10ph)	Activation	Aurora B kinase catalyzes the process, inducing the opening of chromatin condensation regions and promoting transcription factor binding.	Human hepatocellular carcinoma (HCC)
Chromatin remodeling	SWI/SNF complex	Activation	ATP-dependent nucleosome sliding or eviction increases promoter accessibility.	Ovarian cancer, HCC
	PRC1/2	Inhibition	Gene silencing is maintained synergistically through H2AK119ub1 (PRC1) and H3K27me3 (PRC2).	Leukemia, solid tumor
Non-coding RNA regulation	miRNA (miR-21)	Inhibition	Binding to the 3’ UTR of target gene mRNA inhibits translation or promotes degradation.	Glioma, lung cancer
	circRNA (CDR1as)	Activation	Sequestering miR-7 to relieve its suppression of target genes (e.g., EGFR/RAF1), then activating specific signal pathways.	Colorectal cancer, glioma
	LncRNA (XIST)	Inhibition	Recruiting the PRC2 complex to the X chromosome mediates H3K27me3 deposition and chromosome silencing	Turner syndrome

## 3 Epigenetic gene editors and editing tools

Due to the evident biological effects of epigenetic regulation, the idea of making use of epigenetic modification to obtain specific effects came into birth, which boost the development of epigenetic editing tools. Here we are going to introduce some epigenetic editors.

### 3.1 Gene editing

Gene editing, also referred to as genome editing or genome engineering, is the process of modifying the genome of a specific organism through the utilization of gene editing technology. Following the identification of the first element of CRISPR (Clustered Regularly Interspaced Short Palindromic Repeats)/Cas9 (CRISPR-associated protein 9) in 1987, the application of the CRISPR/Cas9 system to bacteria began in 2012, leading to a significant surge in related research. Consequently, gene editing technology has become increasingly sophisticated, with a proliferation of gene editors (20). The following types of gene editing tools are currently available: (1) CRISPR/Cas9 system: The CRISPR/Cas9 system was the earliest discovered, and is also the most widely used gene editing technology at present. It consists two components, i.e., clusters of regularly spaced short palindromic repeats (CRISPR) and related protein (Cas9). Cas9 protein is a type of nucleic acid endonuclease composed of 1,409 amino acids and contains two key structural domains: the crossover junction endodeoxyribonuclease RuvC domain and the HNH domain. CRISPR is a DNA sequence consisting of highly conserved repeat sequences and spacer sequences. The workflow of the system is outlined as follows: the transcribed and processed sgRNA (single guide RNA) folds into a specific three-dimensional structure to form a complex with Cas9 nucleic acid endonuclease, which directs the enzyme to recognize a specific target site and cut the DNA double strand at the neighboring motif of the original spacer sequence, i.e., upstream of the PAM (protospacer-associated motif) sequence. The HNH domain is responsible for cutting the DNA single strand complementary to the sgRNA, which is located 3 nucleotides upstream of the PAM sequence, while the RuvC domain is responsible for cutting the other DNA strand, which is located 3–8 nucleotides upstream of the PAM sequence (21). (2) ZFN technology, otherwise referred to as zinc finger nuclease technology, is a specific type of gene editing technology that utilizes a zinc finger protein to target and modify specific DNA sequences. As the nomenclature implies, this system comprises a zinc finger protein (ZFP) and Fok1 endonuclease. The ZFP comprises a recognition domain that identifies a specific site and binds to it, while the endonuclease executes a shearing function. These two components work in tandem to cleave the double-stranded DNA at the target site. The broken DNA fragments can then be repaired by homologous recombination or non-homologous end-joining repair mechanisms. It is notable that homologous recombination repair may be accompanied by insertion or restoration modifications, while non-homologous end-joining repair mechanisms can result in deletion or insertion mutations. It is important to note that both of these repair mechanisms can result in code-shifting mutations, thereby achieving the objective of gene editing and gene knockout (22, 23). (3)Transcription activator-like effector nucleases (TALEN) technology: TALENs are composed of TALE and Fok1 endonuclease; TALE is also known as a transcription activator-like effector, which is a set of tandem arrays of DNA-binding sequences that can specifically recognize and target the binding DNA site, while Fok1 endonuclease performs the same function as described above, i.e., completing the shearing of the DNA double strand. Subsequent to this, the insertion, deletion and fusion of specific sequences are accomplished according to the intracellular repair mechanism to repair the damage (24). (4)Base editing technology: This emerging field of gene modification technology is predicated on the CRISPR/Cas9 system and is primarily categorized into two distinct classifications: adenine base editing (ABE) and cytosine base editing (CBE). The two technologies are fundamentally similar, with the exception of the different target bases (25, 26). Both rely on the DNA localization ability of CRISPR/Cas to locate a specific base deaminase to a specific position in the genome. This process catalyzes the deamination of a specific base (e.g., cytosine C or adenine A), which is then transformed into another base (e.g., uracil U or hypoxanthine I). During the process of DNA replication, U is treated as thymine T and I as guanine G, thus facilitating the conversion of C to T or A to G (27, 28).

### 3.2 Epigenetic editing

In contrast to the gene editing technologies previously referenced, epigenetic editing, founded on the principles of epigenetics, facilitates precise regulation of gene expression and protein levels without compromising the DNA sequence of an organism (29). The historical development of epigenetic editing technology can be traced back to the mid-20th century, particularly following the proposal of the concept of epigenetics. The seminal contributions of British developmental biologist Conrad Hal Waddington, who first proposed the concept of epigenetic inheritance in 1942, and of Arthur Riggs and Robin Holliday, who in 1975 proposed that methyl modification of DNA can affect gene expression, represent a major breakthrough in the field of epigenetics. Subsequent to these seminal contributions, and alongside the development of molecular biology technology, epigenetics underwent a more systematic research phase. Notable epigenetic phenomena encompass DNA methylation, genomic imprinting, maternal effect, and gene silencing, among others. The study of these phenomena not only deepened the understanding of epigenetic inheritance, but also provided a theoretical basis for the development of epigenetic editing technology. The discovery of the CRISPR/Cas9 system led to the rapid development of gene editing technology. Subsequent to this, scientists modified the Cas9 protein so that it lost the enzymatic activity of cutting DNA but retained the function of binding to sgRNA and localizing to target DNA sequences. This development gave rise to the early epigenetic editing technology, CRISPR-dCas9 technology. Since then, the field of epigenetic editing has developed rapidly, with a proliferation of novel technologies, which have in turn provided new ideas and methods for disease treatment, gene function research, and precision medicine (30).

### 3.3 Epigenetic editing tools

Despite the recent advent of epigenetic technologies, a number of types of epigenetic editors have already been produced. CRISPR-dCas9 technology is a gene editing variant based on the CRISPR-Cas9 technology. Mutations in two key structural domains, RuvC and HNH, in the Cas9 protein result in the loss of the nucleic acid endonuclease activity of the Cas9 protein, thereby transforming it into a dCas9 protein. However, the dCas9 protein still retains the function of binding to sgRNA and localizing to the target DNA sequence. The CRISPR-dCas9 technology facilitates the composition of a variety of epigenetic editors through the combination of distinct structural domains or proteases. These editors can be categorized into two distinct groups: inhibitory epigenetic editors and activating epigenetic editors. The two categories of editors, respectively participate the formation of CRISPRi (CRISPR inhibition technique) and CRISPRa (CRISPR activation technique). The distinction between these two categories is determined by the expression of activating/repressing genes (31) of evolutionary engineering and synthetic biology methodologies (32).

#### 3.3.1 Inhibitory epigenetic editor

##### 3.3.1.1 KRAB

The Krüppel associated box (KRAB) domain is a category of transcriptional repression domains present in approximately 400 human zinc finger protein-based transcription factors (KRAB zinc finger proteins) (33). It has been established that approximately 350 protein-coding genes in the human genome contain the KRAB structural domain, which has a transcriptional repressive effect (31). The dCas9-KRAB fusion protein was constructed by Gilbert et al. by fusing the dCas9 protein with the KRAB structural domain. Subsequent studies revealed that dCas9-KRAB exerts its repressive effect on target genes by recruiting the methyltransferase SETDB1 (SET Domain Bifurcated Histone Lysine Methyltransferase 1) to the target site (34). In addition to this, dCas9-KRAB has been shown to be capable of targeting gene regulatory elements. For instance, dCas9-KRAB has been observed to target the HS2 enhancer, increase modifications to enhancer H3K9me3, decrease enhancer and promoter chromosome accessibility, and silence the expression of multiple bead protein genes (35). Consequently, dCas9-KRAB fusion proteins are currently the most widely used tools for gene suppression, gene function studies, gene regulatory element screening, and disease therapeutic screening (36–38). In addition to the inhibition of gene expression, dCas9-KRAB fusion proteins have the capacity to induce DNA methylation in order to explore the epigenome. Furthermore, dCas9-KRAB has been employed to devise innovative strategies for the treatment of hepatocellular carcinoma, alcoholic fatty liver disease, metastatic cancers, and lymphomas by regulating histone modifications and gene transcription (39–42). Additionally, dCas9-KRAB can be utilized to develop methods for the identification of gene regulatory elements. Gasperini et al. have combined CRISPR with the expression of quantitative trait loci to present the CRISPR quantative trait locus (CRISPRQTL) mapping, a framework for expressing multiple quantitative trait loci. The framework employs dCas9-KRAB to disrupt 5,920 candidate enhancers in cells, followed by single-cell RNA sequencing. The application of this framework has enabled the identification of 644 cis-enhancer gene pairs and 471 high-confidence enhancer gene pairs, thereby underscoring the potential of CRISPRQTL mapping in facilitating large-scale mapping of enhancer gene regulatory interactions (43). Among the identified KRAB structural domains, the zinc finger imprinted 3 (ZIM3) KRAB structural domain has been identified as a very potent repressor. It has been demonstrated to be more effective in target gene silencing, and is less sensitive to gRNA (guide RNA) selection than currently available systems. Furthermore, the smaller construct of ZIM3 KRAB provides an advantage for the utilization of viral delivery methods that are limited by insertion length (44). Pattali et al. fused DNMT3A, DNMT3L, and KRAB to the dCas9 protein to construct a CRISPRoff structure (DNMT3A-DNMT3-dCas9-KRAB). Transient CRISPRoff expression has been shown to lead to highly specific DNA methylation and to maintain gene repression through cell division and differentiation in stem cells. Furthermore, the epigenetic silencing induced by CRISPRoff is not limited to genes with typical CpG islands, but also silences genes which do not possess such islands. The broad and stable gene silencing exhibited by CRISPRoff in the genome is attributed to its targeting of numerous promoters and enhancers from diverse genes. This finding suggests that CRISPRoff induces a stable epigenetic memory. In comparison to other CRISPRi tools, CRISPRoff offers a more extensive range of options for studying the effects of gene methylation modifications on gene function and the regulation of flexible gene expression (45).

##### 3.3.1.2 DNMT

DNA methyltransferase (DNMT) is responsible for catalyzing the process of CpG island DNA methylation, thereby exerting a regulatory effect on gene expression (46). Researchers have developed a dCas9-DNMT3A fusion protein, comprising the attachment of the dCas9 protein to the catalytic structural domain of DNMT3A. This fusion protein has been shown to induce DNA methylation and repress gene expression in the promoter regions of the IL6ST (interleukin 6 signal transducer) and BACH genes. However, the off-target effect of the dCas9-DNMT3A fusion protein limits the assessment of DNA methylation. To address this limitation, a SunTag array has been fused to the dCas9 protein to construct the dC9Sun-D3A system, which uses dCas9-SunTag to recruit proteins to target sites. This system has been shown to independently regulate the expression of the DNMT3A catalytic structural domain and dCas9-SunTag, thereby facilitating more accurate and efficient DNA methylation editing. Furthermore, dCas9-DNMT3A fusion proteins have been utilized to elucidate disease mechanisms and identify potential therapeutic targets. Furthermore, research on DNMT is currently being utilized in the fields of drug development and the preclinical stages of disease treatment (47). The DKK3 gene, which encodes a secreted protein known as DKK3, has been shown to inhibit the growth and metastasis of prostate tumors. In many cancers, DKK3 promoter methylation results in the downregulation of its expression. Gene silencing studies have shown that DKK3 maintains normal prostate epithelial cell homeostasis by restricting TGF-β/Smad signaling. A study utilizing the DNA methyltransferase (DNMT) inhibitor decitabine to treat PC3 prostate cancer cells resulted in the demethylation of the DKK3 promoter, leading to elevated DKK3 expression and the inhibition of TGF-β/Smad-dependent transcriptional activity. This finding provides a foundation for potential therapeutic approaches in the treatment of prostate cancer (48).

##### 3.3.1.3 HDAC

Histone deacetylase (HDAC) is the primary catalyst of the deacetylation process *in vivo*, which has the capacity to inhibit the expression of target genes by decreasing the level of histone acetylation in gene regulatory regions. Like other CRISPR tools, dCas9-HDAC can induce genomic deacetylation, which is used to study the effects of epigenetic modifications on biological processes. A research team has reconstructed the dCas9-HDAC8-EGFP fusion and performed histone deacetylation of the promoters of ESR1 (EStrogen Receptor 1), TERT (Telomerase reverse transcriptase), and CDKN1C (Cyclin-dependent kinase inhibitor 1C) genes in cancer cell lines MCF-7 (Michigan Cancer Foundation-7), and MDA-MB-231 (M D Anderson—Metastatic Breast—231), and HEK 293 (Human embryonic kidney 293) cells. The results demonstrated that dCas9-HDAC8-EGFP, in conjunction with specific gRNAs, effectively repressed the expression of ESR1, TERT, and CDKN1C genes by specifically depleting the level of H3K9ac at the recruitment site. Conversely, the results of dCas9-HDAC8-EGFP-induced epigenetic editing were neutralized upon the use of HDAC inhibitors. In addition to the down-regulated gene expression effect, the cellular response to estradiol and tamoxifen treatment was altered accordingly due to the epigenetic editing of the ESR1 gene by dCas9-HDAC8-EGFP (49). The dCas9-HDAC fusion protein has been used in further studies to target the DPP4 promoter, reducing histone acetylation, inhibiting DPP4 (Dipeptidyl peptidase-4) expression and significantly reducing tumor growth and metastasis. This suggests a potential therapeutic strategy for chromatin remodeling in metastatic cancer (50).

#### 3.3.2 Activating epigenetic editor

##### 3.3.2.1 Herpes simplex viral protein 64

VP64, a tetramer of herpes simplex virus 16 (VP16), has been shown to activate gene expression at the transcriptional level. In addition, dCas9-VP64 has been demonstrated to significantly enhance gene expression when guided by single or multiple sgRNAs targeting NTF3 (Neurotrophin 3) and VEGFA (Vascular Endothelial Growth Factor A), suggesting that this fusion protein can specifically activate the expression of endogenous human genes. dCas9-VP64 is a potent CRISPR-activated tool that has been extensively utilized to study the activation of several genes, disease mechanisms, and other aspects (51). For instance, a specific long non-coding RNA (lncRNA), designated GRASLND, was identified as a regulator of cartilage formation by differentiating mesenchymal stem cells. Silencing of GRASLND led to a reduced accumulation of cartilage-like extracellular matrix, whereas overexpressing GRASLND (via endogenous activation of CRISPR-dCas9-VP64) significantly enhanced the production of cartilage matrix. This finding suggests that GRASLND plays a significant role in regulating cartilage formation in stem cells and that it has potential therapeutic benefits in the treatment of cartilage-related diseases, such as osteoarthritis (52). Hepatic stellate cells play a crucial role in the development of hepatic fibrosis, and thus have become a specific therapeutic target for antifibrotic therapy. The targeted induction of hepatic stellate cells into hepatocytes by delivering clusters of regularly interspaced short palindromic repeats (CRISPR-dCas9) is expected to treat liver fibrosis. It has been demonstrated that the CRISPR/dCas9-VP64 system, when encapsulated within alpha mouse liver 12 cell (AML12) derived exosomes, can be effectively delivered into hepatic stellate cells. In turn, the CRISPR/dCas9-VP64 system loaded in the exosomes could be efficiently released into hepatic stellate cells. This provides a strategy for gene therapy of liver fibrosis (53). Recent studies have revealed that methyltransferase-like 3 (METTL3), a pivotal RNA N6-adenosyl methyltransferase, exhibits substantial upregulation in human hepatocellular carcinoma (HCC) and multiple solid tumors. Furthermore, METTL3 expression has been associated with a poor prognosis in patients with HCC. The CRISPR/dCas9-VP64 activation system has been employed to demonstrate that METTL3 expression significantly promotes HCC growth both *in vitro* and *in vivo*, thereby facilitating a deeper understanding of the mechanisms underlying epigenetic alterations in liver carcinogenesis (54).

##### 3.3.2.2 p300

The p300 protein is an important histone acetyltransferase that activates gene expression and is involved in a variety of biological processes. dCas9-p300 has been widely used to screen and characterize target gene regulatory elements because of its efficient and specific transcriptional activation. dCas9-p300, like other CRISPR/dCas9 regulatory tools, can help to study the effect of elements on genes. Like other CRISPR/dCas9 regulatory tools, dCas9-p300 can also be used to study the effects of elements on genes (55). Cystic fibrosis transmembrane conductance regulator (CFTR) is a membrane protein and anion channel in vertebrates that is encoded by the CFTR gene. The CFTR gene codes for an ABC transporter-class ion channel protein that conducts chloride and bicarbonate ions across epithelial cell membranes. The regulatory mechanisms of CFTR genes in different tissues are diverse and complex, and are realized by multiple regulatory elements. However, how these regulatory elements regulate CFTR gene expression is unclear. The team of Kababi selected 18 high-priority regions and targeted them with dCas9-p300 and dCas9-KRAB to assess their ability to regulate CFTR expression. The results suggest that increasing CFTR expression using dCas9-p300 may improve the efficacy of therapeutic modulators and contribute to the discovery of new therapeutic interventions for the treatment of cystic fibrosis (CF) (56); The dCas9-p300 platform is a powerful tool for studying acetylation modifications in biological processes, and some investigators have used dCas9-p300 or dCas9-HDAC8 fusion proteins to mimic or block acetylation induced by Fos gene enhancer activity to study how histone acetylation regulates Fos gene transcription through transient and rapid changes. Increased histone acetylation prolonged Fos gene transcription time and ultimately increased Fos protein levels; (36) In the context of gene therapy for colorectal cancer, it was found that ZNF334 is a newly discovered member of the zinc finger structure and that aberrant epigenetic reprogramming of the promoter region of the ZNF334 gene reduced its expression in colorectal cancer and further induced colorectal carcinogenesis, In the study, the CRISPR/dCas9-p300 system increased histone acetylation in the ZNF334 promoter region, which normalized the defects in ZNF334 expression to inhibit colorectal cancer growth, providing a promising gene therapy strategy for the treatment of colorectal cancer (57).

##### 3.3.2.3 VPR

dCas9-VPR is a gene activation tool based on the CRISPR-Cas9 system that achieves upregulated expression of specific genes by fusing inactivated Cas9 (dCas9) with the transcriptional activation domain VPR. In a related study of telomeres, progressive telomere shortening was found to be caused by TERT deficiency, leading the researchers to conclude that ectopic overexpression of the TERT gene was a strategy to achieve cellular immortalization. In this work, the researchers reactivated the endogenous TERT gene in unstimulated peripheral blood T cells by epigenetically labeling the promoter of TERT using transcriptional activators (VPH and VPR) based on the CRISPR-Cas9 system, and succeeded in delaying their cellular senescence by at least 3 months. This work provides new ideas for a deeper understanding of the mechanisms of cellular senescence (58); PTEN (Phosphatase and tensin homolog) is an important multifunctional tumor suppressor gene that inhibits a wide range of cellular processes, including survival, cell cycle progression and migration; loss of PTEN activity contributes to the development of many malignancies, which are associated with poor prognosis and the development of drug resistance. One study used the CRISPR-dCas9-VPR system to target the PTEN proximal promoter via sgRNA to cancer cells with low levels of PTEN expression and found that the dCas9-VPR system increased PTEN expression in melanoma and triple-negative breast cancer cell lines. Activation of PTEN significantly inhibited downstream oncogenic pathways, including AKT, mTOR, and MAPK signaling. CRISPR-mediated targeted activation of PTEN may provide an alternative therapeutic approach to the current treatment of refractory and highly aggressive cancers (59).

##### 3.3.2.4 Protein arginase methyltransferase

Protein arginase methyltransferase (PRMT) is an epigenetic target with clinical potential. It can methylate proteins (both histones and non-histone proteins) (60). To date, nine members of the PRMT family, named PRMT1-9, have been identified, with highly homologous SAM-dependent methyltransferase (MTase) catalytic structural domains and distinct motif structures outside the catalytic structural domains. PRMT1 and PRMT6 contain only a single MTase domain; PRMT2, PRMT3, PRMT4, PRMT5, PRMT8, and PRMT9 all have N-terminal motifs before the catalytic domain; and PRMT7 and PRMT9 both contain duplicated MTase domains (61). PRMT can transfer methyl groups from S-adenosine methionine to the guanidinium nitrogen atoms of the arginine side chain of the protein to produce methylated arginine. There are three forms of arginine methylation that can be regulated by PRMT: monomethylarginine methylation, asymmetric dimethylarginine methylation, and symmetric dimethylarginine methylation. Based on these three types of regulation, PRMTs are divided into three major categories: type I, II, and III. Type I consists mainly of PRMT1, PRMT2, PRMT3, PRMT4, PRMT6, and PRMT8, which catalyze the formation of asymmetrically dimethylated arginine from the substrate; type II, consisting of PRMT5 and PRMT9, catalyzes the formation of symmetrically dimethylated arginine from the substrate; and type III, consisting only of PRMT7, is responsible for catalyzing the formation of monomethylated arginine from the substrate. PRMT plays an important role in key cell cycle processes such as regulation of DNA repair, cell cycle progression, transcriptional regulation and RNA splicing (62). Previous studies have suggested that PRMT is a potential oncogene and that overexpression of PRMT plays a pro-oncogenic role in colorectal, lung, ovarian, prostate, pancreatic, lymphoma, leukemia, and glioblastoma. PRMT inhibitors therefore represent a new strategy for treating tumors at the genetic level (63).

##### 3.3.2.5 Histone lysine demethylase

Histone lysine demethylase (HKDM) is divided into two classes: one class is lysine-specific demethylase (LSD), which is dependent on flavin adenine dinucleotide (FAD) for its function, including histone LSD1, also known as KDM1. The human KDM1 family includes KDM1A and KDM1B, which catalyze the demethylation of histone H3K4me1/2 methylation. The KDM1 family has been implicated in epigenetic regulation and has been shown to play important roles in various biological processes and disease pathogenesis, including cell differentiation, embryonic development and hormone signaling. In addition, not only is there a strong association between aberrant KDM1A expression and the development of various types of cancer, but analysis of clinical data also suggests that KDM1A expression is closely associated with tumor lymph node staging, distant metastasis and poor prognosis. Because KDM1A is elevated in various tumor types, it is considered an important tumor oncogene. Therefore, many pharmacological inhibitors of KDM1A have been developed and shown to inhibit tumor cell proliferation, invasion and migration and are being tested as candidates for cancer therapy (64). Another class of HKDMs is the Jumonji C domain-containing protein (JMJD) family of histone demethylases, which includes KDM2-7. For example, the KDM5 family consists of four members, KDM5A, KDM5B, KDM5C, and KDMD, all of which are histone H3K4me2/3 demethylases containing the JumonjiC structural domain. In breast cancer cells, KDM5A is significantly elevated at both the mRNA and protein levels. Post-translational modifications (e.g., phosphorylation, methylation, and ubiquitination) of the KDM gene have a significant impact on its function. These modifications can alter the subcellular localization, stability, enzymatic activity, and interactions with other proteins of KDM, thereby affecting its role in breast cancer and other diseases. The PI3K (phosphatidylinositol 3-kinase)/AKT (Protein Kinase B) signaling axis is often overactive in breast cancer cells and leads to cancer progression. Inhibition of the PI3K/AKT pathway reduced H3K4me3 levels and decreased the expression of cell cycle regulatory genes in various breast cancer cells. Further studies showed that KDM5A is a target of AKT and that phosphorylation of KDM5A by AKT increased its localization in the cytoplasm while decreasing its binding to chromatin, and since the phosphorylation status of KDM5A determines its subcellular localization, this suggests that modulating the PTM of KDM5A may be a promising anticancer strategy (65).

#### 3.3.3 Evolved engineered transcriptional repressor

Evolved engineered transcriptional repressor (EvoETR) technology is an evolutionary engineering and synthetic biology approach to the optimization and design of gene transcriptional regulatory networks. EvoETR is based on the principle that DNA binding domains (DBDs) and effector domains are contained in the same molecule to target and regulate the expression of specific genes. DBDs are specifically designed to bind precisely to specific DNA sequences in the genome, ensuring that they can be accurately targeted to the target gene. Common DBDs include zinc finger proteins (ZFPs), transcriptional activation-like effectors (TALEs) and dCas9 (catalytically inactivated Cas9) in the CRISPR-dCas9 system, all of which can be used to direct EvoETR to a specific gene locus. The effector domains (EDs) is the part responsible for performing the gene silencing function and is usually derived from naturally occurring transcriptional repressors. In EvoETR, EDs can inhibit gene expression by introducing repressive epigenetic marks, such as histone methylation, into the promoter region of the target gene through mechanisms such as the recruitment of histone modifying enzymes. Representing a new class of epigenetic editing technologies, EvoETR technology can be precisely localized to the promoter region of the target gene, ensuring specificity of gene silencing. In addition, once EvoETR introduces repressive epigenetic marks, these marks can persist in the cell, resulting in permanent gene silencing. In addition, compared to CRISPR-dCas9 technology, EvoETR technology has reduced off-target effects and significantly improved targeting and safety. At present, EvoETR technology has a wide range of potential applications in the treatment of genetic diseases, cancer and other diseases. For example, by targeting and silencing disease-related genes, it is possible to treat or alleviate diseases. In particular, EvoETR has shown significant results in the treatment of hypercholesterolemia and related cardiovascular diseases. A parallel study by Capelluti et al. reported the use of an evolved engineered transcriptional repressor (EvoETR) system to silence the PCSK9 (Proprotein convertase subtilisin/kexin type 9) gene involved in the control of low density lipoprotein (LDL) levels, consisting of a gene associated with the DNMT3A catalytic structural domain and its cofactor DNMT3L fused to ZFP. The researchers found that EvoETR was able to silence the PCSK9 gene in a highly specific and durable manner, thereby reducing plasma levels of PCSK9 protein and thereby regulating cholesterol metabolism, providing a new strategy for the treatment of hypercholesterolemia and related cardiovascular diseases.

**FIGURE 2:**
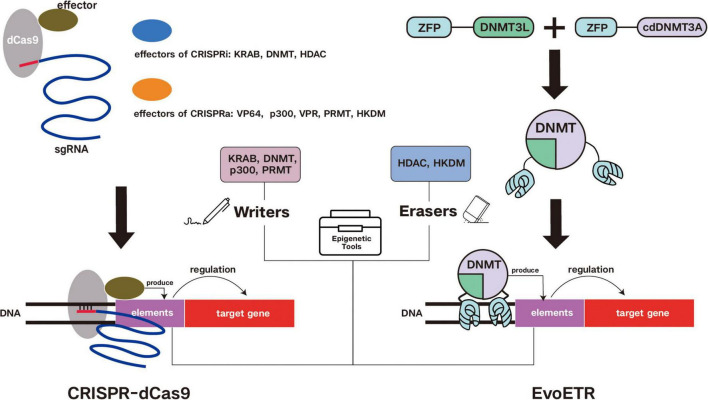
Mechanisms of commonly used epigenetic editor. Frequently used epigenetic editors include CRISPRa and CRISPRi, which utilize the CRISPR-dCas9 system coupled with diverse modification elements, and EvoETR (Evolutionarily Engineered Transcriptional Repressors). These editors can be categorized as “Writers” or “Erasers” based on their function of adding or removing modifications to target genes, respectively. Writer elements (e.g., KRAB, DNMT, p300, and PRMT) introduce covalent modifications to target genes, whereas eraser elements (e.g., HDAC and HKDM) remove such modifications. While CRISPR-dCas9-based epigenetic editors rely on the CRISPR-dCas9 system for target recognition, EvoETR employs zinc finger proteins (ZFPs) for targeting and utilizes the DNMT3A catalytic domain to execute epigenetic modifications on target genes.

Figure 2 illustrates the basic mechanisms of the commonly used epigenetic editing tools introduced earlier.

## 4 Bottlenecks and optimization of epigenetic editors

Though epigenetic editors have been deeply developed, there are still various bottlenecks in current technologies.

### 4.1 Delivery

The blood-brain barrier (BBB) presents a major obstacle to the delivery of macromolecules into the brain. Therefore, the safe and efficient transport of epigenetic gene editors to specific cell types within the brain is crucial for the success of epigenetic editing therapies targeting neurological disorders (66–68). Existing delivery strategies are generally categorized into viral and non-viral vectors.

#### 4.1.1 Viral vectors delivery

Through viral vectors is a frequently used method to import epigenetic editing tools into cells, mainly including adeno associated virus (AAV) and lentivirus. These viral vectors exhibit distinct characteristics in terms of delivery efficiency, targeting specificity, and safety, offering diverse options for the application of epigenetic gene editing technology in the treatment of neurological disorders.

The most frequently used viral vector is AAV. It has unique biological characteristics making it one of the ideal vectors to deliver epigenetic editing tools. AAV has high safety, and natural AAV is non-pathogenic to the human body. After modification, some of its own genes are removed, further reducing potential risks and minimizing the impact on the normal physiological functions of host cells. It also has a good tissue specificity, meaning that it can infect specific cell types. For instance, AAV is able to infect neurons and neuroglia cells efficiently. By choosing different serotypes, we can make AAV target on different brain areas and cell types. For example, AAV9 serotype can efficiently cross the blood-brain barrier and is widely distributed in brain and spinal cord tissues, providing convenience for the treatment of neurological diseases (66). However, AAV still has some disadvantages. Its packaging capacity is limited, usually only able to accommodate exogenous gene fragments of no more than 4.7 kb, which limits the use of some larger epigenetic editing tools, such as some CRISPR based DNA editing technologies like the CRISPRoff system, a synthetic sgRNA that fragments in response to light, preventing formation of new double strand breaks (DSBs) (69). Neumann point out that the D3A-D3L-dCas9-KRAB fusion fragment, which constitutes CRISPRoff, is approximately 6.2 kb in length. This significantly exceeds the packaging capacity of the AAV vector, which is about 4.7 kb (including inverted terminal repeats) (70). This limitation poses challenges for efficient delivery to target cells, thereby hindering the achievement of gene editing. When applying related gene-editing technologies to the treatment of central nervous system diseases, the oversized CRISPRoff system faces significant challenges in effective delivery via AAV vectors, which are the preferred tool for transgene delivery to the central nervous system. This limitation hinders its application in this field. Moreover, AAV may initiate immune reactions, causing vectors eliminated and influencing the treatment effect (66). When injecting AAV carrying epigenetic editing tools into the brain of animal models, it may trigger an immune response in the body, producing antibodies against AAV, thereby reducing the circulation time and delivery efficiency of the vector *in vivo*.

Lentivirus is a genus of retrovirus, which has unique advantages in delivering epigenetic editing tools. It can infect both dividing and non-dividing cells, including neurons, glial cells, making it have a wide range of applications. In the study of neurological diseases, lentiviruses can effectively deliver epigenetic editing tools to different types of nerve cells, achieving regulation of related genes. For example, delivering CRISPR-dCas9 based DNA methylation editing tools to midbrain substantia nigra dopaminergic neurons through lentivirus can successfully regulate the expression of SNCA genes, reduce the aggregation of α-synuclein, and provide a potential therapeutic approach for Parkinson’s disease. The genome of lentivirus can integrate into the host cell’s genome, enabling long-term stable expression of the target gene (66). This is particularly crucial for the treatment of neurological diseases, as many of these conditions are chronic and require sustained gene regulation to maintain therapeutic effect. Compared to some other viral vectors, lentivirus induces relatively weaker immune responses, especially in *in vitro* experiments, causing minimal interference with host cells. This characteristic allows lentivirus to reduce issues such as vector elimination and diminished therapeutic effect caused by immune responses when delivering epigenetic editing tools *in vivo*. However, lentivirus is still not a perfect vector. Its random integration into the host genome may lead to insertional mutations, potentially activating oncogenes or disrupting tumor suppressor genes, which may pose a risk of cancer (66). So it is essential to strictly monitor and evaluate integration sites to decrease this risk when using it for gene therapy. Although lentivirus has stronger packaging capacity than some other viruses, it still has limitations in applications requiring the delivery of larger gene fragments. It takes time for lentivirus to integrate into the genome, resulting in potentially lower transient expression efficiency compared to viruses that do not integrate into the genome. In certain *in vivo* applications, lentivirus may still trigger immune responses, affecting delivery efficacy. Additionally, it is sensitive to environmental conditions and prone to inactivation, which makes it storage and use under strictly controlled conditions.

#### 4.1.2 Non-viral vectors

Non-viral vectors include nanoparticle delivery systems and polymeric delivery systems.

Lipid nanoparticles (LNPs) are a commonly used non-viral vector in nanoparticle delivery system with unique advantages in delivering epigenetic editing tools. They are mainly composed of phospholipids, cholesterol, PEG-lipids, and ionizable lipids. Under certain conditions, these components can self-assemble into nanoscale particles, which effectively enhance the stability of DNA or RNA, protecting them from degradation by nucleases. LNPs can be taken up by cells through various pathways, such as endocytosis. Once entering the cell, LNPs are transported to endosomes, where the ionizable lipids become protonated, causing structural changes in the lipid nanoparticles. This facilitates their fusion with the endosomal membrane, releasing the encapsulated DNA or RNA into the cytoplasm (67). In research on the treatment of neurological diseases, LNPs have been utilized to deliver epigenetic editing tools. For instance, in a study of Alzheimer’s disease, LNPs carrying a CRISPR-dCas9-based epigenetic editing system were delivered to the brains of mice. The experimental results demonstrated that LNPs effectively transported the editing tools to neurons and glial cells in the brain, partially regulating the expression of related genes and reducing Aβ protein deposition (67). However, the targeting capability of LNPs is relatively limited, typically relying on passive targeting mechanisms, such as the enhanced permeability and retention (EPR) effect in tumor or diseased tissues, to achieve drug accumulation. In neurological diseases, the presence of the blood-brain barrier makes it difficult for LNPs to specifically target diseased cells in the brain. Additionally, the stability and biodistribution of LNPs *in vivo* are influenced by various factors, such as serum protein adsorption and immune system recognition. These factors may lead to the elimination of LNPs before they reach target cells, affecting their delivery efficiency.

Polymeric delivery systems represent another important kind of non-viral vectors, utilizing electrostatic interactions between polymers and nucleic acids to complete nucleic acid delivery. Commonly used polymers include cationic polymers such as polyethyleneimine (PEI) and polylysine (PLL). These cationic polymers contain positive charges, enabling them to bind with negatively charged DNA or RNA through electrostatic interactions, forming nanoscale complexes (68). Taking PEI as an example, it is a highly branched cationic polymer containing numerous amino groups. Under physiological conditions, these amino groups become partially protonated, giving PEI a positive charge. When PEI is mixed with DNA or RNA, the positively charged PEI and the negatively charged nucleic acids bind together through electrostatic attraction, forming stable complexes. The surface charge, particle size, and morphology of these complexes can be controlled by adjusting factors such as the polymer-to-nucleic acid ratio and reaction conditions. Polymeric delivery systems display excellent biocompatibility, as many polymers can be gradually degraded *in vivo*, consequently reducing potential toxicity to the organism. The structure and properties of polymers can be regulated through chemical modifications, achieving the optimization of nucleic acid delivery efficiency, targeting specificity, and stability. For instance, by modifying the polymer surface with targeting ligands such as antibodies or peptides, targeted delivery to specific cell types can be done. In research on neurological diseases, modified polymeric delivery systems have been employed to deliver epigenetic editing tools. By attaching peptides to the polymer surface that specifically recognize receptors on neuronal cells, targeted delivery to neurons has been achieved. Experimental results show that these modified polymeric delivery systems can effectively transport epigenetic editing tools into neurons and regulate related genes within these cells (68).

However, both nanoparticle delivery systems and polymeric delivery systems still have limitations as vectors. Although nanoparticle has the advantage of small size in crossing the blood-brain barrier, the barrier’s high selectivity and tight junction structure still make it hard for nanoparticle to effectively cross it and reach the brain parenchyma. The distribution and metabolic processes of nanoparticles *in vivo* are not yet fully researched, and their long-term safety and potential toxic side effects require further investigation. To polymeric delivery systems, some cationic polymers may exhibit toxicity at high concentrations, impairing normal cellular functions. Furthermore, polymeric delivery systems generally have relatively low delivery efficiency, particularly in the complex physiological environment of the body, where they may be disrupted by factors such as competitive binding with serum proteins and recognition by the immune system (68).

#### 4.1.3 Optimization of delivery system

In order to optimize the delivery system, researchers are exploring various strategies. One of the ideas is to reform and optimize existing vectors. For example, modifying the capsid proteins of AAV to change their surface properties can enhance their affinity for neurons and improve delivery efficiency (66). By introducing targeting ligands onto the AAV capsid proteins, they can specifically bind to receptors on the surface of neurons, enabling targeted delivery to these cells (67, 68). Additionally, we can develop novel delivery vehicles, such as nanomaterial-based carriers, which have excellent biocompatibility and modifiability. These carriers can achieve targeted delivery to specific cell types through surface modifications. Smart nanocarriers constructed using nanotechnology can precisely release editing tools in response to microenvironmental changes at the disease site, such as pH, temperature, and enzyme activity, thereby enhancing therapeutic efficacy (68).

### 4.2 Off-target effects

Off-target effects represent an important technical challenge for epigenetic editing tools, significantly impacting the safety and efficacy of treatments. Current epigenetic editing tools, such as those based on the CRISPR-Cas system, exhibit high specificity but still face challenges in completely eliminating off-target effects (71). This limitation arises because the CRISPR-Cas system depends on the complementary pairing of guide RNA (gRNA) with the target DNA sequence to identify specific sites. However, gRNA can non-specifically bind to off-target genomic regions that resemble the target sequence, resulting in unintended modifications by the Cas protein at these sites (71). These off-target effects may alter the expression of non-target genes, potentially disrupting normal cellular physiological functions. In Alzheimer’s disease treatment research, off-target effects of epigenetic editing tools can result in unintended modifications to genes essential for normal brain function, potentially triggering new neurological dysfunctions (72). Additionally, off-target effects may pose potential carcinogenic risks. If off-target modifications occur in regions associated with tumor suppressor genes or oncogenes, they could alter the expression levels of these genes, disrupting the normal regulatory mechanisms of cell growth and then increasing the risk of cancer. In Parkinson’s disease treatment research, if epigenetic editing tools cause off-target modifications to genes involved in the regulation of cell proliferation and apoptosis, they may disrupt the normal balance of neuronal growth and death, leading to abnormal cell proliferation and increasing the risk of nervous system tumors (73).

To mitigate off-target effects, researchers have adopted multiple strategies. Optimizing the design of gRNA is a crucial approach. By using bioinformatics analysis to select gRNA sequences with high specificity to the target sequence and low complementarity to off-target sequences, the likelihood of gRNA binding to off-target sites can be reduced (74).

### 4.3 Cytotoxicity epigenetic

Editing tools, especially CRISPR-based editing systems, although possessing enormous potential in gene regulation, also face cytotoxicity issues (71). For instance, the DNA methyltransferase (D3A) used in the CRISPRoff system can cause cytotoxicity when continuously expressed, particularly when combined with zinc finger proteins (ZFP), where this toxicity becomes more pronounced. Transient overexpression of ZFPoff constructs containing the D3A catalytic domain exhibited significant cytotoxicity in HEK293T cells, while cells transfected with the same ZFP fusion without this catalytic domain recovered quickly. This cytotoxicity may stem from prolonged expression of editing tools and non-specific targeting effects. Additionally, in AAV vector-based gene editing, the concatemers formed by AAV genomes can express transgenes long-term, potentially causing bacterial enzymes (such as Cas9) to become antigenic over time, triggering immune responses that lead to cytotoxicity (75). For example, when AAV vectors are used to deliver CRISPR editing tools, they may induce long-term immune responses *in vivo*, thereby increasing cytotoxicity. Moreover, a large proportion of the human population already has immune memory against Cas9, further increasing the risk of immune reactions. Long-term expression may also lead to increased off-target activity, interfering with normal gene expression regulation and affecting normal cellular functions (75).

To reduce the cytotoxicity of epigenetic editing, researchers have proposed various optimization strategies. Among these, an epigenetic editor called CHARM (Coupled Histone Tail for Autoinhibition Release of Methyltransferase) developed by Neumann et al. has significant advantages in this aspect. It achieves methylation and silencing of target genes by recruiting endogenous DNMT3A through the combination of histone H3 tail and non-enzymatic DNMT3L C-terminal domain (D3L). This method avoids using the catalytically active D3A domain, thereby reducing cytotoxicity. By using smaller zinc finger proteins (ZFP) instead of the CRISPR system, CHARM can be successfully packaged into a single AAV vector, which not only improves delivery efficiency but also reduces immune responses and cytotoxicity caused by multi-vector delivery (70). Furthermore, CHARM editors can also turn off the expression of the editor by targeting the promoter in the AAV vector. This self-silencing mechanism can adjust the expression time of the editor as needed, reducing cytotoxicity while achieving therapeutic effects (70). Additionally, to address the potential risks of cytotoxicity from long-term expression, researchers are exploring short-term expression strategies, such as using “hit-and-run” technology to rapidly clear gene editing tools from cells after completing the editing task, avoiding immune responses and off-target risks caused by long-term presence (76). Simultaneously, developing new gene editing tools that act transiently in cells, such as mRNA-based editors, which rapidly degrade after editing, reduces the continuous impact on cells.

### 4.4 Editing efficiency and stability

Improving the efficiency and stability of epigenetic editing represents a significant technical challenge currently faced (76). In the treatment of various diseases, it is essential to ensure that epigenetic editing tools can efficiently target specific genes and that these editing effects can be maintained stably over the long term.

In terms of editing efficiency, although current epigenetic editing tools have achieved some effects in certain experiments, there remains a problem of relatively low efficiency. When delivering CRISPR-dCas9-based epigenetic editing tools to neuronal cells, the complexity of the intracellular environment and limitations of delivery vectors make it difficult for editing tools to effectively enter cells and reach target gene location, thereby affecting editing efficiency (74). In some studies on neurodegenerative diseases, although changes in target gene expression can be detected, the magnitude of these changes is small and insufficient to produce significant therapeutic effects. This may be due to uneven distribution of editing tools within cells, or degradation or inactivation before reaching the target sites. Editing stability is also a key issue. Epigenetic modification is a dynamic process, and cells have multiple mechanisms to maintain the balance of epigenetic states. Therefore, modifications introduced by epigenetic editing tools may be affected by intrinsic cellular mechanisms, leading to unstable editing effects (76, 77). In somatic cells, gene silencing effects induced by some KRAB-based epigenetic editors are unstable. Cappelluti et al. pointed out that due to the instability of KRAB-associated histone marks in somatic cells, long-term gene silencing effects are difficult to maintain unless chromatin-bound repressors are continuously deposited (78). This means that continuous expression of editors is required to maintain the repressed state of genes, increasing the complexity and potential risks of treatment. Additionally, during cell replication, some epigenetic editing effects may be difficult to maintain, failing to ensure stable transmission of the post-edited epigenetic state to daughter cells, resulting in weakened or lost gene silencing effects. This poses a significant challenge for therapeutic strategies that require long-term maintenance of gene editing effects, limiting their application in the treatment of diseases that require sustained suppression of specific gene expression.

To improve editing efficiency and stability, researchers have conducted multifaceted explorations. In delivery systems, they have developed new vectors to enhance the delivery efficiency and cellular uptake of editing tools. For example, vectors constructed with nanomaterials exhibit good biocompatibility and targeting ability, enabling more effective delivery of editing tools to neuronal cells (77). In the design of editing tools, researchers have optimized their structure and function to enhance binding capacity to target genes and modification activity. By modifying the dCas9 protein, they have enabled tighter binding to target gene loci and improved the efficiency of recruiting epigenetic modifying enzymes (74). Simultaneously, by studying intracellular epigenetic regulatory networks, researchers have gained understanding of factors affecting editing stability and developed corresponding strategies to maintain editing effects. For instance, by inhibiting cellular demethylase activity, they reduce the removal of newly added methyl groups, thereby stabilizing the methylation state of genes; through researching histone modification marks or DNA methylation patterns that can be stably transmitted during cell division, and by designing corresponding editors, they introduce these stable epigenetic marks to target gene location, achieving long-term stable gene silencing or activation (76, 77).

### 4.5 The complexity and applicability of disease treatment

Many diseases, such as cancer and neurodegenerative disorders, involve multiple genes and complex cellular processes in their pathogenesis (71, 77). Extensive cancer-related research has highlighted the complexity of cancer development and the necessity of multi-gene regulation. For example, cancer genomics studies suggest that single epigenetic editing may be insufficient to fully address the intricate gene expression abnormalities underlying these diseases. Cancer progression, in particular, is driven by mutations in multiple genes, epigenetic alterations, and disruptions in cellular signaling pathways (71). Thus, silencing or activating a single gene alone is often inadequate for effective treatment. Achieving optimal therapeutic outcomes requires the precise and simultaneous regulation of multiple genes. However, current technologies face significant challenges in implementing coordinated multi-gene editing.

Additionally, different cell types exhibit variable responses to epigenetic editing. Zhang et al. reported that while the PPAD-dCas9 editor successfully manipulates gene transcription in various mammalian cells, the degree of gene upregulation varies across cell lines, with some gene regulation showing cell-type dependency (79). This variability complicates the selection of appropriate cell types for editing specific diseases and limits the broader application of epigenetic editing technologies.

To address the complex mechanisms underlying various diseases, future research should prioritize the development of multi-gene cooperative editing technologies. For instance, the multi-target editing capabilities of the CRISPR-Cas system can be utilized to precisely regulate multiple disease-related genes simultaneously. By designing optimized sgRNA combinations, researchers can achieve the concurrent silencing, activation, or modification of multiple genes, thereby enabling more effective treatment of complex diseases (71). Regarding the differential responses of cell types to epigenetic editing, personalized cell-type adaptation strategies should be explored. In-depth analyses of gene expression profiles, chromatin states, and other cell-specific characteristics can facilitate the creation of cell-type-specific editing databases to guide gene editing for particular cell types. Furthermore, optimizing the design and delivery methods of gene editing tools based on the unique features of different cell types can enhance both editing efficiency and applicability.

## 5 Progress in treating human disease with epigenetic editors

Nowadays, researchers are trying to use epigenetic editors in the treatment of varieties of diseases, bringing new choices and hope to the patients of some refractoriness diseases.

### 5.1 Hypercholesterolemia

**FIGURE 3:**
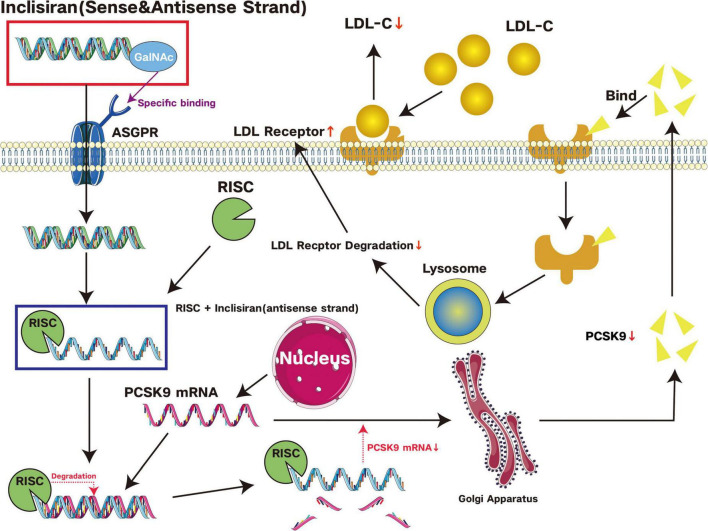
Schematic representation of Inclisiran’s mechanism of action. Inclisiran is a siRNA therapeutic agent incorporating N-acetylgalactosamine (GalNAc) moieties. GalNAc, a carbohydrate ligand, selectively binds to asialoglycoprotein receptors (ASGPRs) expressed abundantly on hepatocyte plasma membranes. ASGPRs facilitate glycoprotein endocytosis, enabling efficient hepatocyte-specific delivery of Inclisiran through GalNAc-ASGPR interaction. Upon cellular internalization, Inclisiran’s sense strand degrades while its antisense strand associates with the RNA-induced silencing complex (RISC). This complex targets PCSK9 mRNA for degradation, subsequently diminishing PCSK9 protein synthesis. PCSK9 is a kind of protein that regulates the amount of low-density lipoprotein receptor (LDLR). When bound to LDLRs, it promotes their lysosomal degradation, decreasing LDLR density on hepatocyte surfaces. By suppressing PCSK9 expression, Inclisiran increases hepatic LDLR concentration, enhancing low-density lipoprotein cholesterol (LDL-C) clearance from circulation and consequently reducing plasma LDL-C levels significantly.

Hyperlipidemia is a condition in which abnormalities in the metabolism or function of lipids cause human blood lipid levels to exceed the normal range, as evidenced by high blood cholesterol and/or triglycerides or low HDL. PCSK9 is the ninth member of the kexin-like pre-translational enzyme family of Bacillus subtilis proteases, which are proteins that bind to the LDL receptor and induce the degradation of LDL. The LDL receptor functions in the body to remove LDL from the blood and transport it to the liver for metabolism. When the activity of PCSK9 is increased, it leads to a decrease in the number of LDL receptors, which reduces the efficiency of LDL removal and causes blood cholesterol levels to rise. Since the discovery of the extreme mechanism of action of PCSK9 at the beginning of this century, the development of PCSK9 inhibitors has become a hot topic (80). Current PCSK9 inhibitors include alirocumab and evolocumab (81). Evolocumab is a PCSK9 monoclonal antibody developed by Amgen. It is a fully human monoclonal IgG2 that reduces circulating low-density lipoprotein cholesterol (LDL-C) levels by binding to PCSK9 and preventing it from binding to the low-density lipoprotein receptor (LDLR), thereby reducing LDLR degradation. Alirocumab, a PCSK9 inhibitor co-developed by Sanofi and Regenerative Elements, is a fully human monoclonal IgG1. By inhibiting circulating PCSK9, alirocumab prevents PCSK9-mediated degradation of LDLRs, thereby increasing the number of LDLRs on the cell surface and lowering serum LDL-C levels. Alirocumab and evolocumab are both now FDA-approved and can be taken alone or in combination with a lipid-lowering agent. In addition to the two approved monoclonal antibodies, Novartis has also approved a small interfering RNA (siRNA) drug. Inclisiran is a double-stranded siRNA that degrades PCSK9 mRNA in the liver and blocks PCSK9 protein synthesis. This increases recirculation and expression of the LDL-C receptor on the surface of hepatocytes, increasing hepatic uptake and lowering circulating LDL-C. Inclisiran’s mechanism of action also involves its prolonged presence in hepatocytes after clearance from plasma, allowing for a prolonged duration of LDL-C lowering effects. The efficacy of a single injection can be maintained for half a year, making it an ultra-long-acting PCSK9 inhibitor. It is indicated for the treatment of primary hypercholesterolemia (heterozygous familial and non-familial) or mixed dyslipidaemia in adults and in patients who do not achieve LDL-C treatment goals despite treatment with the maximum tolerated dose of statins and in patients for whom statins are intolerable or contraindicated (82). The Figure 3 gives a brief schematic diagram of the mechanism of Inclisiran.

### 5.2 Carcinoma

Cancer is still the leading cause of death that threatens human life and health. Basically, cancer is a disease caused by changes in the genome, mainly due to DNA mutations. These mutations can lead to the inactivation of oncogenes, which can weaken or eliminate the inhibitory effect on tumors; they can also lead to the activation of proto-oncogenes into oncogenes and the transformation of normal cells into tumor cells. The process by which normal cells undergo genetic and epigenetic changes leading to the gradual development of malignant characteristics is known as cancer epigenetics. In recent years, studies of the human cancer epigenome have provided insights into the molecular pathways of cancer development, and epigenetic editing techniques are now being successfully used to explore new therapeutic strategies for cancer. Below are some examples of research advances in epigenetic editors in cancer therapy (83).

#### 5.2.1 Bladder carcinoma

Bladder cancer is a prevalent malignancy of the urinary system, characterized by high morbidity and mortality rates globally. Approximately 75% of patients present with non-muscle invasive bladder cancer (NMIBC), while 25% are diagnosed with muscle-invasive bladder cancer (MIBC). KDM6A (Lysine Demethylase 6A), an enzyme responsible for the demethylation of H3K27me2/3, exhibits a high mutation frequency in bladder cancer. Notably, approximately 50% of NMIBC patients and 25% of MIBC patients harbor inactivating or deleterious mutations in KDM6A. Research has demonstrated that KDM6A functions as a tumor suppressor by inhibiting the proliferation, migration, and invasion of bladder cancer cells, and its expression is strongly correlated with patient prognosis. KDM6A exerts its anti-tumor effects through the epigenetic activation of RHGDIB transcription, which suppresses Rac1—a key regulator of tumor cell motility, invasiveness, and metastasis. Furthermore, the FOXA1-KDM6A-ARHGDIB axis has been identified as a critical pathway in bladder cancer metastasis, underscoring the therapeutic potential of targeting KDM6A in clinical settings. Beyond KDM6A, other genes involved in epigenetic regulation, including KMT2C (Lysine N-methyltransferase 2C), KMT2D (Lysine N-methyltransferase 2D), and ARID1A (AT-rich interactive domain-containing protein 1A), also display aberrant expression patterns in bladder cancer. To date, only a few small molecule compounds targeting histone modifiers in bladder cancer have entered clinical trials, and most are still in phase I/II research. Among these, HDAC inhibitors are the most studied epitopes in bladder cancer clinical trials. However, results from clinical trials suggest that for patients with locally advanced or metastatic bladder cancer, epitope-only therapeutic strategies have limited efficacy and are often associated with significant toxic side effects. Based on preclinical studies, we have found that targeting histone modifying factors has a significant modulating effect on the tumor immune microenvironment (84). Epimedicines in combination with immunotherapy have shown satisfactory therapeutic effects in several preclinical bladder cancer models. As a result, several clinical trials have been initiated and preliminary results show that most patients have a good response and tolerability to this combination regimen. This suggests that the combination of epimedicines with classical therapies (e.g., chemotherapy, immunotherapy, etc.) may be a superior therapeutic strategy (84).

#### 5.2.2 Prostatic carcinoma

Prostate-specific antigen (PSA) is a proto-oncogene that is specifically highly expressed in embryonal carcinoma cells and prostate cancer cells, whereas it is barely expressed in normal prostate tissue cells. The specific expression of PSA was found to be related to the conditional transcriptional regulation of its promoter. CRISPR-dCas9-KRAB is a newly developed transcriptional regulatory system that inhibits gene expression by interrupting the DNA transcription process. The researchers constructed a CRISPR-dCas9-KRAB system driven by the PSA promoter that inhibits PSA gene expression in prostate cancer cells at the transcriptional level, thereby suppressing malignant growth and migration of cancer cells and promoting their apoptosis. This study provides a potentially effective anti-cancer strategy for prostate cancer gene therapy (85); Prostate cancer development is also associated with other epigenetic regulation, in terms of DNA methylation and demethylation, DNMT1 has oncogenic activity in the early stage of prostate cancer, while it has oncogenic activity in the late stage, promoting metastasis by inducing epithelial-mesenchymal transition and a cancer stem cell phenotype. Aberrant methylation of DNMT1 is associated with adverse clinicopathological types and survival, e.g., DNMT1 regulates steroid 5-alpha reductase 2 (SRD5A2), and methylation of its promoter region is associated with improved survival in patients with castration-resistant prostate cancer (CRPC) treated with ADT (86–89). TET enzymes oxidize 5 mC–5 hmC, and the TET family of proteins promotes site-specific DNA demethylation in normal cells, but in prostate cancer, its tumor suppressor TET family proteins are inhibited, such as TET2, which is involved in Androgen Receptor (AR) signaling, and its down-regulation is associated with prostate cancer progression, TET proteins can inhibit tumor progression and invasion by down-regulating the methylation of key genes, and it is considered as a new prognostic biomarker along with 5 hmC (90–92). In terms of histone modification, JMJD1A, as a key coactivator of AR, is epigenetic genetic regulation H3K9 methylation recruits Heterogeneous Nuclear Ribonucleoprotein F (HNRNPF) to promote variable splicing of AR-V7, and silencing of JMJD1A reduces AR-V7 levels; JMJD2A drives prostate carcinogenesis through the transcription factor ETS Variant Transcription Factor 1 (ETV1). Lysine-Specific Demethylase 1 (LSD1) acts as a transcriptional repressor of AR-regulated enhancers through H3K4 and H3K9 demethylation, interacts with RCOR1 (REST corepressor 1)/CoREST and phosphorylates H3Thr-6 as an AR coactivator to promote Castration-Resistant Prostate Cancer (CRPC) progression, Targeting LSD1 in combination with AR antagonists is a promising approach for the treatment of CRPC and LSD1 inhibitors are being clinically investigated in prostate and other cancers (93); PRC2 is a multi-complex protein consisting of Enhancer of Zeste Homolog 2 (EZH2) and Embryonic Ectoderm Development (EED), etc., and EZH2 overexpression is associated with prostate cancer progression, which can be inhibited by microRNA101. In the ADT-induced neuroendocrine prostate cancer (NEPC) mouse model, EZH2, and CBX2 (Chromobox 2) are highly expressed, and EZH2 overexpression is a driver of NEPC progression, and in the MYCN (V-myc myelocytomatosis viral related oncogene)-induced NEPC mouse model, EZH2 and MYCN co-inhibit AR signaling to drive NEPC development, and EZH2 is a key therapeutic target (94–96).

#### 5.2.3 Lymphoma

Lymphomas are hematological tumors. Among them, peripheral T-cell lymphomas (PTCL) are a rare and heterogeneous group of clinically aggressive diseases. With the exception of ALK + interstitial large cell lymphoma (ALCL), most peripheral T-cell lymphomas are highly malignant with an aggressive course, poor clinical outcome, poor remission rates and frequent relapses after first-line therapy. Histone deacetylase (HDAC) is often aberrantly expressed in peripheral T-cell lymphomas, causing disease progression and leading to a poor prognosis. Low levels of HDAC7 and HDAC1/2 activity are required for the development of T-cell lymphomas. In addition, inactivation of HDAC3 leads to significantly lower levels of expression of most genes, affecting cell cycle progression and functional T-cell transformation, and overexpression of HDAC6 is also associated with poor outcome. Potential associations between HDAC or EZH2 expression and the prognosis of PTCL subtypes have also been investigated and have shown that EZH2 and HDAC1/2 are frequently upregulated in patients with PTCL and that patients with higher EZH2 and HDAC2 expression typically have poorer survival (97). Cedarbromide is a selective HDAC inhibitor that inhibits cell proliferation and blocks cell cycle progression in the G0/G1 phase. In addition, cedarbromide inhibits the phosphorylation levels of proteins in the AKT/mTOR and MAPK signaling pathways and activates the DNA Damage Response (DDR) cell cycle checkpoint pathway (ATM-Chk2-p53-p21 pathway) in lymphomas. Cedarbenamide is currently approved by the FDA for the treatment of peripheral T-cell lymphoma. In addition to cedarbenamide, romidepsin, and belistat are also FDA-approved HDAC inhibitors for the treatment of peripheral T-cell lymphoma. In addition to HDAC inhibitors, two DNMT inhibitors, azacitidine and decitabine, have been approved for clinical use by the FDA and the European Medicines Agency. Tazemestat, an EZH2 inhibitor that has shown superior methyltransferase activity in EZH2-mutant versus wild-type Follicular Lymphoma (FL) in early clinical trials, has also been granted accelerated approval by the FDA for the treatment of R/R FL. Tazemestat in combination with rituximab and in combination with lenalidomide and rituximab for the treatment of relapsed/refractory FL remain in clinical trials (98).

**FIGURE 4:**
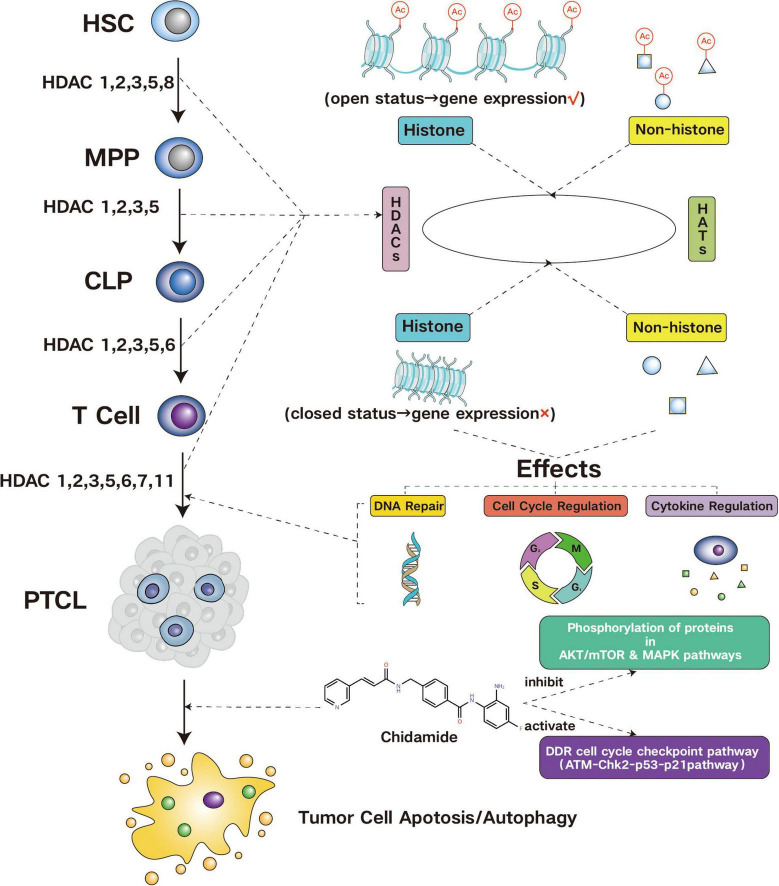
The association between HDAC and peripheral T-cell lymphoma (PTCL). Histone deacetylases (HDACs) mediate the deacetylation of both histone and non-histone substrates. In histones, HDAC-mediated deacetylation induces chromatin condensation, leading to transcriptional repression. HDACs also play crucial roles in DNA repair, cell cycle regulation, and cytokine modulation, among other biological processes. Moreover, various HDAC isoforms are essential for T-cell development. These mechanisms suggest that abnormal HDAC expression in peripheral T-cells contributes to the onset and progression of PTCL, resulting in poor prognosis. Vorinostat, a selective HDAC inhibitor, exerts therapeutic effects in PTCL by inhibiting the phosphorylation of key proteins in the AKT/mTOR and MAPK signaling pathways, while also activating the DNA damage response (DDR) cell cycle checkpoint pathway (ATM-Chk2-p53-p21 pathway). This leads to apoptosis or autophagy in lymphoma cells.

The association between HDAC and PTCL and a brief introduction to the mechanism of Chidamide is shown in Figure 4.

### 5.3 Neurological diseases

Neurological disorders represent a significant threat to human health, placing substantial burdens on patients, their families, and society. Common conditions such as Alzheimer’s disease and Parkinson’s disease. Current clinical treatment strategies for these disorders remain limited, relying primarily on medications, physical therapy, and rehabilitation training. While medications can mitigate symptoms to some extent, they are unable to cure the underlying conditions and may produce adverse effects with prolonged use. Physical therapy and rehabilitation primarily aim to improve functional impairments and enhance patients’ quality of life but provide limited efficacy in addressing the root causes of these diseases. Epigenetic gene editing technology has shown tremendous potential in neurological disorder treatment by modulating disease-associated gene expression, repairing damaged neural cell functions, and even promoting neural cell regeneration and repair. Advancing research into the application of epigenetic gene editing in neurological disorders is of substantial theoretical and practical significance, offering promising new therapeutic avenues and renewed hope for patients.

#### 5.3.1 Alzheimer’s disease

Alzheimer’s disease (AD) is a prevalent neurodegenerative disorder predominantly affecting older adults, with incidence rates increasing markedly with age. The pathologies include extracellular β-amyloid protein (Aβ) aggregation forming amyloid plaques and intracellular hyperphosphorylation of Tau protein resulting in neurofibrillary tangles. These pathological alterations induce neuronal dysfunction and death, culminating in progressive cognitive deterioration and substantially diminished quality of life. Epigenetic dysregulation plays a fundamental role in AD pathogenesis and progression. Aberrant DNA methylation patterns have been identified within promoter regions of numerous AD-associated genes in affected brain tissues. A critical target in this context is postsynaptic density protein 95 (PSD95), encoded by the Dlg4 (Disks large homolog 4) gene, which serves essential functions in neuronal plasticity and memory formation. As the predominant scaffold protein in excitatory postsynaptic densities, PSD95 orchestrates interactions among various neuronal components including synaptic glutamate receptors, signaling molecules, adhesion proteins, cytoskeletal elements, and other scaffolding proteins. Studies indicate PSD95’s crucial role in postsynaptic organization, plasticity regulation, and neural circuit stabilization (99–101). Notably, PSD95 expression becomes dysregulated during AD progression, suggesting that its modulation may ameliorate cognitive impairments—providing a theoretical foundation for epigenetic editing as a therapeutic approach.

In investigating epigenetic mechanisms underlying AD pathogenesis, Bustos and colleagues engineered zinc finger proteins (ZFPs) specifically targeting the Dlg4/PSD95 gene promoter. These ZFPs were conjugated with various effector domains to generate artificial transcription factors or epigenetic modulators. The constructs included PSD95-6ZF-VP64 designed to enhance Dlg4/PSD95 expression, while PSD95-6ZF-G9a, PSD95-6ZF-Suvdel76, and PSD95-6ZF-SKD were engineered to repress it. These molecular tools function by modifying histone marks to regulate transcription. The researchers subsequently introduced these PSD95-6ZF fusion constructs into mouse models via viral vector delivery to evaluate their effects on hippocampal neurons. Experimental findings revealed that PSD95-6ZF fusion constructs effectively modulated synaptic and dendritic spine maturation in hippocampal neurons, influencing excitatory synaptic transmission. Particularly noteworthy, PSD95-6ZF-VP64 transduction significantly upregulated PSD95 expression and successfully rescued memory deficits in the AbPPswe/PS-1 mouse model of AD. This intervention produced significant improvements across multiple cognitive parameters, including novel object recognition (NOR), object location memory (OLM), and spatial learning performance (72).

#### 5.3.2 Prion disease

Prion diseases, also termed Transmissible Spongiform Encephalopathies (TSEs), constitute a group of fatal neurodegenerative disorders caused by misfolded prion proteins (PrP). The primary pathogenic agent, the abnormal isoform of prion protein (PrPsc), induces the conversion of normal cellular prion protein (PrPC) into PrPsc. This process results in PrPsc accumulation in the central nervous system, ultimately causing neuronal damage and death. PrPsc aggregation within neurons initiates a cascade of cytotoxic effects. Studies demonstrate that PrPsc aggregates disrupt normal neuronal functions, interfering with intracellular signaling pathways and impairing mitochondrial activity. The amyloid fibrils and oligomers formed by PrPsc aggregation exhibit neurotoxicity, compromising cell membrane integrity, disrupting ion homeostasis, and inducing oxidative stress and inflammatory responses. These pathological changes accelerate neuronal damage, leading to cell death and loss, which ultimately manifest as neurological dysfunction characterized by hallmark symptoms such as ataxia and cognitive impairment. Genetic factors play a crucial role in prion disease pathogenesis, with approximately 10–15% of cases being familial and strongly linked to PRNP gene mutations. The PRNP gene encodes the prion protein, and specific mutations (e.g., D178N, E200K) alter its structure and function, increasing its propensity for abnormal folding and aggregation, thereby elevating disease risk. Furthermore, various PRNP mutations contribute to distinct clinical phenotypes and disease progression patterns (102–104).

**FIGURE 5:**
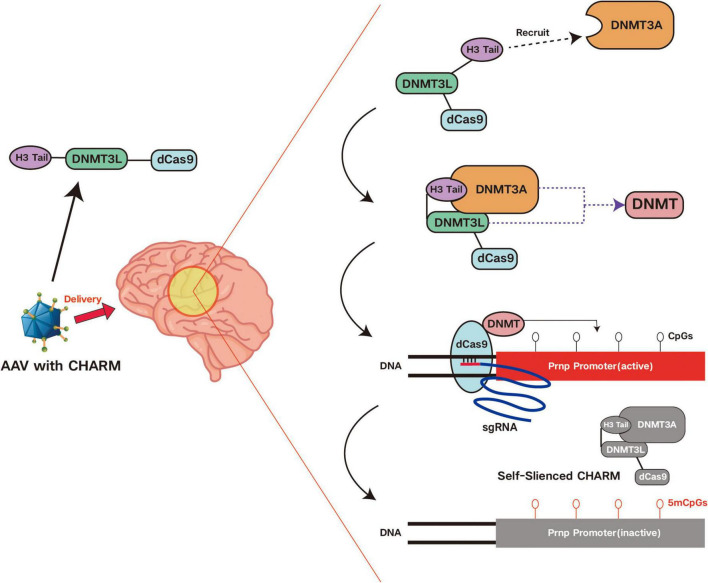
Mechanism of action of CHARM. CHARM is an innovative, compact, enzyme-independent epigenetic editor. Using an AAV vector, CHARM is delivered to the brain to target the PRNP gene. It produces promoter methylation of the PRNP gene by directly fusing the histone H3 tail with the DNMT3L domain, which recruits and activates endogenous DNA methyltransferases, silencing gene transcription.

Neumann’s research team explored prion disease pathogenesis using an epigenetic editing strategy based on DNA methylation. They developed a novel editor, CHARM (Coupled Histone tail for Autoinhibition Release of Methyltransferase), which effectively reduced prion protein levels in mouse brains, demonstrating its therapeutic potential. CHARM functions as a compact, enzyme-free epigenetic editor by directly fusing the histone H3 tail with the Dnmt3l domain, recruiting and activating endogenous DNA methyltransferases to methylate target genes and silence their transcription. This design circumvents the overexpression of potentially cytotoxic catalytic domains, minimizing cellular toxicity. By delivering CHARM to mouse brains via an adenoviral vector targeting the PRNP gene, the researchers achieved widespread gene silencing and methylation. Experimental outcomes revealed a 70–90% reduction in PRNP transcripts and a 60–80% decrease in PrP protein levels without significant side effects. As an innovative epigenetic editing tool, CHARM demonstrates considerable potential for treating prion diseases and offers promising therapeutic opportunities for patients. This technology may also advance treatment strategies for other neurodegenerative disorders (70). Figure 5 shows the mechanism of CHARM briefly.

## 6 Discussion

Taking epigenetics as the starting point, this paper first reviews the core concept of epigenetic inheritance and summarizes its four major mechanisms. Among these, DNA methylation—a covalent modification of DNA—is the most extensively studied. According to current statistics, DNA methylation plays a crucial role in disease development, response to environmental exposures, development and differentiation, and biomarker discovery. In clinical practice, it is mainly used in the diagnosis of tumors, including early diagnosis and screening of tumors, prognostic risk assessment and therapeutic efficacy evaluation.

Histone phosphorylation has also emerged as a significant research focus in recent years. With technological advancements from hardware to software, mass spectrometry-based proteomics has enables large-scale detection from routine biological samples to single-cell and even spatially resolved analyses, greatly improving the depth and accuracy, and structural insights of protein identification and quantification. Together with the discovery of disease biomarkers and the development of marker detection methods, proteomics shows great potential in the process of translation into clinical applications, and we believe that today, with the impetus of automation, multi-omics data integration and, in particular, artificial intelligence, there will be even greater breakthroughs in this field.

The role of chromatin remodeling and regulation of non-coding RNAs in epigenetic processes cannot be ignored. Over 20% of cancer patients carry variants in genes that encode chromatin remodeling proteins, and some cancers are caused entirely by mutations in these genes. This highlights the critical role of chromatin remodeling in tumorigenesis and cancer progression. Moreover, chromatin remodeling proteins are required for the growth and survival of a wide range of tumors. This suggests that chromatin remodeling proteins are potentially important drug targets. Therefore, an in-depth understanding of the mechanism of action of chromatin remodeling proteins and their regulatory networks is crucial for the development of new tumor therapeutic strategies.

Non-coding RNAs, including lncRNAs, circRNAs and miRNAs play a pivotal role in the development, diagnosis and treatment of certain tumors. Currently, tissue biopsy remains the gold standard for tumor detection. If the diagnosis of ncRNAs can be effectively used in the clinic, ncRNA-based blood or urine tests may spare tumor patients the pain of invasive testing.

Epigenetic editing technologies have created a variety of epigenetic gene editors by integrating epigenetic concepts based on earlier gene editing technologies, which can be categorized as repressive and activating epigenetic editors according to their agonism or inhibition of the transcription process. The advent of CRISPR/Cas9 and related systems has significantly advanced the field by enabling highly specific, reprogrammable gene modifications through base-pair complementarity, enhancing both safety and efficiency. However, the knockdown or knock-in of large DNA fragments may disrupt the genome’s three-dimensional structure. The emergence of CRISPR/dCas9 provides a potential solution. Compared to CRISPR/Cas9, CRISPR/dCas9 activates or represses gene expression through epigenetic modification without affecting the genome sequence, avoiding the unpredictable effects and minimizing toxicity to cells. Although CRISPR/dCas9 is considered safe, its clinical adoption remains limited due to challenges related to delivery efficiency and off-target risks. With ongoing improvements in these areas and its precise localization capabilities, CRISPR/dCas9 holds significant potential for gene therapy.

To date, epigenetic editors have shown promising applications in various diseases, including metabolic disorders such as hypercholesterolemia, neurodegenerative diseases like Alzheimer’s, neurodevelopmental disorders, and cancers such as prostate and breast cancer. For example, EE targeting PCSK9 reproducibly induces a CpG island methylation signature leading to complete inhibition of circulating PCSK9 protein in mice. The mouse studies also support the development of single-dose treatments for hypercholesterolemia with levels of LDL-C reduction equal to or greater than approved therapies that require long-term administration. However, further evaluation of the efficacy, tolerability and safety are needed, including *in vivo* studies to examine pharmacodynamic and pharmacokinetic properties of the drugs, as well as their biodistribution and potential toxicological effects. Another example involves the Dlg4 gene, which encodes postsynaptic density protein 95 (PSD95)—a synaptic protein essential for plasticity that organizes glutamate receptors. PSD95 levels decline with age and in neurodegenerative diseases such as Alzheimer’s and Huntington’s. Epigenomic analysis of the hippocampus led to the design of a PSD95-6ZF fusion protein, which successfully bound to the Dlg4/PSD95 motif in rats and modulated PSD95 expression. Importantly, PSD95-ATF restored recognition memory in aged mice and Alzheimer’s disease models. This represents the first study demonstrating the potential of targeted gene regulation to improve memory deficits in neurological disorders. In another example, dCas9-KRAB was used to generate truncated PSA mRNA by inhibiting RNA polymerase slippage, leading to degradation of PSA mRNA and subsequent inactivation of cellular PSA expression.

**FIGURE 6:**
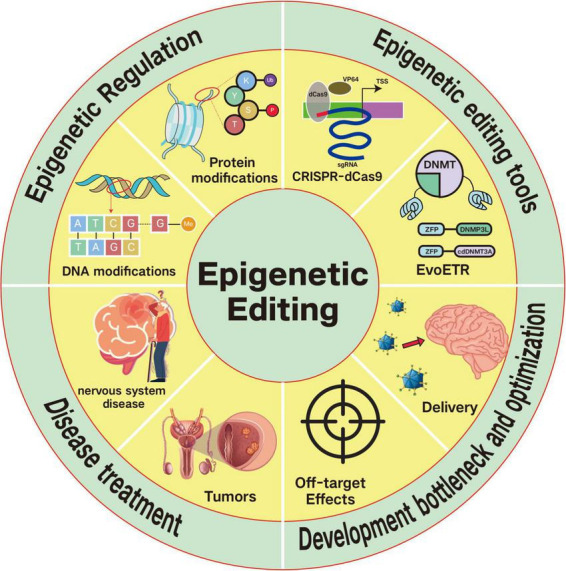
Graphical summary of this article This review examines the core concepts of epigenetics and its underlying mechanisms while highlighting recent advances in epigenetic gene editors and their therapeutic applications. Epigenetics encompasses phenomena that modulate gene expression via chemical modifications of DNA and histones without DNA sequence alteration, including DNA methylation, histone modifications, and non-coding RNA regulation. Current epigenetic editing platforms primarily utilize CRISPR-dCas9-based systems and EvoETR technology. Several challenges impede the clinical translation of epigenetic editing, including delivery limitations, off-target effects, cytotoxicity, insufficient editing efficiency and persistence, and complexities in addressing multigenic disorders. Nevertheless, epigenetic editing approaches have displayed development in treating hyperlipidemia, lymphomas and other malignancies, and neurological conditions such as prion diseases.

In conclusion, we have systematically reviewed the historical background and current advances in epigenetic inheritance. By summarizing recent findings and organizing them around the central concept of epigenetic regulation, we aim to provide a clear and accessible overview that can serve as a useful reference for researchers and clinicians alike. Figure 6 shows the structure and summary of this article.
