# Correction: Precision scalpels for the epigenome: next-gen editing tools in targeted therapies

**DOI:** 10.3389/fmed.2025.1722848

**Published:** 2025-10-27

**Authors:** Ruiming Zhang, Tianqi Yao, Meiyin Fan, Xiaoying Jiang, Keshan Wang, Min Cui, Kaijian Bing, Xiaotian Xia

**Affiliations:** ^1^Department of Nuclear Medicine, Union Hospital, Tongji Medical College, Huazhong University of Science and Technology, Wuhan, China; ^2^Tongji Medical College, Huazhong University of Science and Technology, Wuhan, China; ^3^Health Management Center, Union Hospital, Tongji Medical College, Huazhong University of Science and Technology, Wuhan, China; ^4^Department of Urology, Union Hospital, Tongji Medical College, Huazhong University of Science and Technology, Wuhan, China; ^5^Department of Orthopaedics, Union Hospital, Tongji Medical College, Huazhong University of Science and Technology, Wuhan, China; ^6^Department of Surgical Education Research, Union Hospital, Tongji Medical College, Huazhong University of Science and Technology, Wuhan, China; ^7^Hubei Province Key Laboratory of Molecular Imaging, Wuhan, China

**Keywords:** gene editing, epigenetic editors, cancer target therapy, metabolic disease, neurological diseases

In the published article, the affiliations of **Ruiming Zhang** and **Tianqi Yao** were given only as:

*1.Tongji Medical College, Huazhong University of Science and Technology, Wuhan, China*.

The correct affiliations for both authors should read:

*1.Department of Nuclear Medicine, Union Hospital, Tongji Medical College, Huazhong University of Science and Technology, Wuhan, China;*

*2.Tongji Medical College, Huazhong University of Science and Technology, Wuhan, China*.

The original version of this article has been updated.

